# Whole-Genome Sequencing of the Fungus Penicillium citrinum Reveals the Biosynthesis Gene Cluster for the Mycotoxin Citrinin

**DOI:** 10.1128/MRA.01419-18

**Published:** 2019-01-24

**Authors:** Markus Schmidt-Heydt, Dominic Stoll, Rolf Geisen

**Affiliations:** a Department of Safety and Quality of Fruit and Vegetables, Max Rubner-Institut, Federal Research Institute of Nutrition and Food, Karlsruhe, Germany; Vanderbilt University

## Abstract

Penicillium citrinum is a food-contaminating ascomycete that consistently produces large amounts of the mycotoxin citrinin. Citrinin exhibits, besides its toxicity, antibiotic effects and thus potentially forces antibiotic resistance. Within the genome sequence, we identified the biosynthesis gene cluster for citrinin, which appears to be highly conserved within the genus *Penicillium*.

## Abstract (precis)

Penicillium citrinum is a food-contaminating ascomycete that consistently produces large amounts of the mycotoxin citrinin. Citrinin exhibits, besides its toxicity, antibiotic effects and thus potentially forces antibiotic resistance.

## ANNOUNCEMENT

Penicillium citrinum occurs on salt-rich products, citrus fruits, and wheat and other cereals, the presence of which leads to contamination with the mycotoxin citrinin, and P. citrinum is related to the species P. expansum, P. nordicum, and P. verrucosum (1–3). Citrinin has detrimental effects on the kidneys and the immune system (4). Moreover, the first statin, mevastatin, was isolated from P. citrinum in the 1970s (5).

Genome sequencing was carried out on the MiSeq platform (Illumina) as follows: genomic DNA of P. citrinum strain DSM 1997 was extracted from a pure culture, after growing for 7 days at 25°C on malt extract sucrose agar slants, with the NucleoSpin Plant II kit (Macherey-Nagel) and then quantified and quality checked with NanoDrop 1000 (VWR International) and Qubit 3.0 spectrometers/fluorometers. The sequencing library was built with the Illumina Nextera XT DNA kit and quality checked with the Experion DNA 1K analysis kit (Bio-Rad Laboratories). Raw reads (read length, 2 × 300 bp) were processed with the FastQ preprocessing toolkit (Blast2GO Pro v.5.2). *De novo* assembly was done with SeqMan NGen v.12.3 (Lasergene) with default settings; contigs smaller than 200 nucleotides (nt) and mitochondrial sequences were removed. The assembly size was 31,529,786 bp with 64× coverage and 976 genomic contigs; other parameters were an *N*_50_ value of 67,438 kb, an *L*_50_ value of 137 kb, and a G+C content of 46.15% ± 2.84%. Prediction of biosynthesis gene clusters (BGCs) was carried out with antiSMASH fungal v.3.0 with the cluster-finder algorithm for BGC border prediction with default settings (6, 7).

Twenty-nine BGCs were predicted, 9 type 1 polyketide synthase (T1PKS) clusters, 3 nonribosomal peptide synthetase (NRPS) clusters, 2 polyketide synthase (PKS)-NRPS hybrid clusters, 1 indole-NRPS hybrid cluster, 2 fatty-acid clusters, 1 terpene cluster, and 11 BGCs which were not specified further. Genome annotation based on Aspergillus nidulans was done with AUGUSTUS v.3.0.2 (8), and we identified 9,754 genes.

Within the genome sequence of P. citrinum DSM 1997 a complete BGC for citrinin, showing homology and a high sequence similarity to the citrinin cluster of Monascus purpureus (9), P. expansum, and P. verrucosum (10, 11), was identified. Future analyses of the genome sequence of this food-relevant fungal species will give deeper insight into the secondary metabolite biosynthesis of P. citrinum compared to that of other citrinin-producing fungi.

### Data availability.

The whole-genome sequence of P. citrinum strain DSM 1997 has been deposited at NCBI under the accession number LKUP00000000, and the raw reads were deposited in the SRA under the accession number PRJNA298119.
